# Natural Occurrence of Fungi and Aflatoxins Associated Sugarcane Plant and Sugarcane Juice and Their Control

**DOI:** 10.1007/s12088-023-01171-9

**Published:** 2023-12-30

**Authors:** Marwa A. Younos, E. M. Embaby

**Affiliations:** 1 Food Toxicology and Contaminants Department, Food Industry and Nutrition Research Institute, National Research Centre, Dokki, Giza, Postal Code 12622 Egypt; 2 Plant Pathology Department, Agriculture and Biological Research Institute, National Research Centre, Dokki, Giza, Postal Code 12622 Egypt

**Keywords:** Sugarcane plants, Sugarcane juice, Fungi, Aflatoxins

## Abstract

Sugarcane is one of the most important crops in the world. It is also considered the most popular fresh juice in Egypt. The sugar content of the sugarcane stem represents the main source of fungal growth. This study aimed to investigate the natural co-occurrence of fungi in sugarcane plants and juice, test of aflatoxins production by aflatoxigenic fungi, and improve the quality of sugarcane juice. The obtained results indicated a notable decrease in all physical parameters of the naturally infected sugarcane plants. Isolation of fungi from sugarcane plant and juice from three localities revealed that the highest mean fungal count was recorded in sugarcane rootlets (173.55 cfu/cm), followed by sugarcane stem (94.88 cfu/cm), while sugarcane juice had the least mean fungal count (24.33 cfu/mL). The frequency of the isolated fungi associated with sugarcane plant yielded 781 fungal isolates for rootlets, 427 fungal isolates for stems, and 219 fungal isolates for juice. Four isolates of *Aspergillus parasiticus* were aflatoxins producers. Higher aflatoxin quantity (1434.92 ng/mL) was produced by *A. parasiticus* (isolate No. 21) from sugarcane stem, while *A. parasiticus* (isolate No. 5) from sugarcane juice was less aflatoxins producer (276.95 ng/mL). On the other hand, lemon juice showed a significant reduction effect on the fungal count of peeled and non-peeled sugarcane juice. In which the highest reduction percent of the fungal count was recorded with 20% conc. of lemon on peeled sugarcane juice (36.04%).The obtained results concluded that lemon juice was found to decrease the fungal contaminants and improve the quality of sugarcane juice.

## Introduction

One of the most important crops in the world is sugarcane (*Saccharum officinarum*). Cane sugar makes up around 80% of the sugar consumed worldwide [1, 2]. The sugarcane plant, which has a high sucrose and low fiber content, is mostly used to make raw sugar and molasses as well as bagasse, or leftover grass, which is used as a fertilizer or as a food supplement for animals. In addition, sugar cane and its waste are a major component of numerous secondary industries, including those that produce alcohol, vinegar, paper, chipboard, certain chemicals, fibers, plastics, paints, insecticides, and detergents [3, 4]. One of the most popular fresh juices in Egypt is sugarcane juice, which is available in cane juice shops in every Egyptian city [5, 6]. Sugarcane juice is used in traditional medicine to treat a variety of illnesses like kidney stones, jaundice, lower blood pressure, urogenital tract infections, and healing dermal wounds because of its sweet taste and nutritional value as a source of energy and minerals. It has also been found to have antioxidant properties in various experimental settings [6, 7]. The sugar content in the *S. officinarum* stem represents the main source of fungal growth [8]. Sugarcane is often contaminated simultaneously with several molds which can produce several toxins. Microbes can adhere to surfaces, get inside cells, and grow within the products. Sugarcane juice is popular in many countries as a cheap and sweet beverage. During the harvest season, it is a popular juice that is sold at roadside stands, cafes, and restaurants. However, because of its quick deterioration, the processing and selling of sugarcane juice are restricted [9]. The majority of sugarcane juice street vendors are located next to roadways, the airborne contaminants and microorganisms may taint the juice. *Aspergillus* species are often found in juice and drinking water samples [10–12].Preventing the growth of microbes in the substrate is one of the best strategies to control problems brought on by microbes. Physical treatments include mechanical cleaning, scrubbing, and filtering [13]. The first step from harvest to processing is peeling, which is a crucial step. The process involves removing the top layer of sugar cane along with mud, black spots, and other unwelcome contaminants [14]. The antibacterial properties of lemon, ginger, and salt make them frequent natural preservatives that were initially added to juice to increase flavor. These ingredients also enhance the shelf life of the juice. The antibacterial properties of the natural preservatives are due to their phenolic components [15]. Vitamin C, citric acid, minerals, and phytochemicals like flavonoids, which have many health-promoting characteristics, are just a few of the nutrients that are rich in lemons [16, 17]. The plant's various parts have broad-spectrum antibacterial properties that are effective against both Gram-positive and Gram-negative bacteria as well as *Candida albicans* [18, 19]. This study aimed to investigate the natural co-occurrence of multiple fungi associated with sugarcane plants, the deterioration of sugarcane caused by these fungi, to acquire knowledge of the quality of sugarcane juice that is sold at different places for human consumption in Egypt, isolate a wide variety of fungal species association, to evaluate the occurrence of fungal species and mycotoxins from decayed sugarcane and to improve the quality of sugarcane juice in Egypt.

## Materials and Methods

### Sample Collection

Thirty sugarcane samples were collected from different street vendors and sugarcane juice shops from three different localities (Cairo, Giza, and Qalyubia governorates) in Egypt (10 samples per location) during (2021/2022). Each sample was cut into pieces for easy transporting, and kept separately inside clean sealed sterilized bags, transferred directly to the laboratory, and then kept at 4 °C until immediately processed for mycological and mycotoxin analysis. A natural preservative like lemons was collected from the agricultural farm in the National Research Center, Dokki, Egypt.

### Determination of Deterioration of Naturally Infected Sugarcane Plants (Physical Parameters)

Deterioration of naturally infected sugarcane plants was determined i.e. reduction of plant length (cm), the diameter of size (cm), and volume of juice (mL) compared with healthy plants. Three replicates of each tested sample were used. These attributes of quality are measured by applying principles of physics [20]. Juice is squeezed and measured in mL as the volume of juice was calculated [21].

### Isolation and Purification

#### Mycological Analyses of Sugarcane Plant

Sugarcane billets (stem pieces) were collected and provided by hand. Billet and hand-collected stem pieces at least 3 cm in diameter were selected for sampling; a single piece from each stalk was cut to include the crown roots at the soil surface, and washed with water to remove dust and soil residues. All samples were surface sterilized by 1% sodium hypochlorite for 3 min., washed three times using sterilized water, and aseptically air-dried in a laminar flow hood. Each stem and root was cut into segments (0.5 cm long) by knife, and then each segment was cut into four equal segments. These segments were placed on the surface of the sterilized Potato dextrose agar (PDA) medium containing streptomycin (Three replicates of each tested sample were inoculated with 4 segments for each plate). The inoculated plates were incubated at 26 ± 2 °C for 5 days. The developing fungi were counted as colony-forming units (CFU) per cm for each sample [22].

#### Mycological Analyses of Sugarcane Juice

The sugarcane stalks were washed with water to remove dust and soil residues. The juice was obtained by passing the sugarcane stalks through a power-operated stainless steel roller crusher (SYGA SCJ machine; Power-350 W) after its sterilization using ethyl alcohol 70 %, and then the extracted juice was collected from its outlet. Filtration of juice was done by sieving (1 mm mesh size) and eight layers of muslin cloth to remove debris and insoluble matter. For the observation of fungal flora, a 2 mL sugarcane juice sample was suspended in a sterilized test tube containing 18 mL of sterilized distilled water was shaken well which gave a dilution of 1: 10. 2 mL of suspension from 1: 10 was transferred to the second test tube which gave 1: 100 dilutions. Similarly, 1: 1000 and 1: 10000 dilutions were made. 1 mL aliquot from 1: 10000 dilutions was transferred to the sterilized petri plates with 10 mL of molten cooled Potato dextrose agar (PDA) medium containing streptomycin. The mycoflora of sugarcane juice samples were purified and counted (Three replicates of each tested sample were inoculated with 1 mL for each plate). The inoculated plates were incubated at 26 ± 2 °C for 5 days. The developing fungi were counted as colony-forming units (CFU) per mL for each sample [5, 23].

#### Identification

The pure culture was used for the identification of fungal isolates at the Plant Pathology Dept., National Research Centre (NRC), Egypt. Total fungal count (TFC) and the percentage of fungal frequency were recorded**.**

### Aflatoxin Analysis

#### Aflatoxins Production

All aflatoxigenic fungi (i.e. *Aspergillus flavus* and *Aspergillus parasiticus*) isolates were propagated as pure culture in 100 mL yeast extract sucrose (YES) for testing of aflatoxins production according to Munimbazi and Bullerman [24] and Younos and Akl [25]. Each flask was inoculated with 0.1 mL of spore suspension containing approximately 10^6^ spore's mL^−1^. All cultures were incubated at 26 ± 2 °C for 14 days.

#### Extraction of Aflatoxins from the Culture Media

The extraction was performed according to the procedure offered by Kumar et al. [26] with some modifications as follows. The obtained cultures were filtered and mycelial mats were collected. Aflatoxins were extracted from culture filtrates with chloroform. A known volume of filtrate (25 mL) was added to 10 mL chloroform and was shaken for half an hour. The chloroform contained aflatoxins that were separated by a separating funnel which was allowed to stand for some time until the two layers appeared. The upper aqueous layer was re-extracted many times with chloroform for complete separation. The lower chloroform layer was filtered over anhydrous sodium sulfate in a 250 mL beaker, evaporated in a water bath (70–80 °C) near dryness and the residue was washed twice with chloroform (12 mL) into a glass vial which evaporated till dryness (dry film). The dried extract was kept in the refrigerator at – 5 °C for HPLC analysis.

#### High-Performance Liquid Chromatography (HPLC) Analysis

According to AOAC [27], the HPLC system used was a water 600-pump system equipped with a model 474-fluorescence detector (water) set at 360 nm for excitation and 440 nm for emission wavelength. Water Nova-pack C18 column (150 × 3.9) was used for aflatoxins separation. The mobile phase (water: Acetonitrile: Methanol, 65: 5: 30) has isocratically flowed at a rate of 1.0 mL/min. Data were collected and integrated with a Waters Millennium 32 chromatography Manager software program.

### Control of Fungal Contamination of Sugarcane Juice Using Lemon Juice

Several strategies are available for the decontamination or detoxification of commodities containing mycotoxins.

#### Physical Methods

Physical treatments include cleaning the cane stalk with a knife or motorized peeled, mechanical sorting, separation, and density segregation to prevent the spore and growth of the fungal association. A juice is typically extracted using a simple motorized. Peeled and non-peeled sugarcane juice samples were prepared.

#### Lemon Treatment

Lemon juice was prepared by cutting lemons into pieces with the help of a sharp blade knife. Lemon juice was added to peeled and non-peeled sugarcane juice with different concentrations (2, 5, 10, 15, 20% conc.). One mL aliquot from 1: 10,000 dilutions of sugarcane juice samples was transferred to the sterilized Petri plates and 10 mL of molten cooled agar was poured containing 200 mg/ liters streptomycin. Petri dishes were incubated at 28 ± 2 °C for 5–7 days. Three replicates were used [5]. All formed colonies were counted and recorded.

### Statistical Analyses

Data obtained in this study were analyzed using software (IBM SPSS Statistics v.16. USA). Statistical significance was performed using a one-way Analysis of Variance (ANOVA) test. A value of *P* < 0.05 was considered statistically significant. The least significant difference (LSD) was calculated at *P* ≤ 0.05 according to Gomez and Gomez [28].

## Results

### Determination of Deterioration of Naturally Infected Sugarcane Plants (Physical Parameters)

A notable decrease in all physical parameters of the naturally infected sugarcane plants was observed. Results in Table 1 presented that, plant length (cm) was found to be decreased from 243 cm in the healthy plant to 150 cm in the infected plant with a 38.27 reduction percent in location A sample, as well as decreased from 278 to 144 cm with 48.20% reduction percent in location B sample, while in location C, plant length (cm) was found to be decreased from 256 to 153 cm with reduction percent of 40.23%. Stem diameter (cm) was decreased from 10 in the healthy plant to 6.67 cm in the infected plant with a 33.30% reduction percent in the location A sample, and decreased from 10.13 to 6.57 (cm) with a 35.14% reduction percent in location B sample respectively, while In location C decreased from 10.04 to 6.53 (cm) with reduction percent of 34.96% in the healthy and infected sugarcane plant. Also, the volume of juice/mL was found to be decreased from 983.33 to 208.33 mL with 78.81% reduction percent in location A sample, and decreased from 1166.66 to 221.66 mL with 81.00% reduction percent in location B sample, while in location C decreased from 1100.30 to 218.28 mL with reduction percent of 80.16% in healthy and infected sugarcane plant respectively.

**Table 1:** Determination of deterioration of naturally infected sugarcane plant

Parameter	L	H	I	Loss	%R
Plant height (cm)	A	243 ± 17.00^b^	150 ± 20.00^c^	93	38.27
	B	278 ± 22.91^a^	144 ± 3.61^c^	134	48.20
	C	256 ± 19.95^ab^	153 ± 11.80^c^	103	40.23
Stem diameter (cm)	A	10.00 ± 0.87^a^	6.67 ± 0.42 ^b^	3.33	33.30
	B	10.13 ± 0.50^a^	6.57 ± 0.06 ^b^	3.56	35.14
	C	10.04 ± 0.68^a^	6.53 ± 0.24 ^b^	3.51	34.96
Juice/(mL)	A	983.30 ± 76.38^b^	208.35 ± 14.43 ^c^	775.00	78.81
	B	1166.69 ± 57.74^a^	221.67 ± 10.41 ^c^	945.00	81.00
	C	1100.30 ± 67.06^ab^	218.28 ± 12.42 ^c^	882.02	80.16

*L* Location, *H* Healthy, *I* Infection, Loss = H − I, %*R* Reduction percent. The different letters in each column indicate significant differences at *P* < 05

### Mycological Analyses of Sugarcane Plant

Isolation of fungi from sugarcane plants from three locations revealed that the highest mean fungal count was recorded in sugarcane rootlets (173.55 cfu/cm), followed by sugarcane stem which recorded 94.88 cfu/cm, while sugarcane juice had the least mean fungal count (24.33 cfu/mL). On the other hand, location C had the highest fungal count in sugarcane rootlets (214.66 cfu/cm), followed by locations B and A, which gave 204.66 & 101.33 cfu/cm respectively. In sugarcane stem, location C also had the highest fungal count (129.33 cfu/cm), followed by locations B and A, which recorded 110.66 & 44.66 cfu/cm respectively, while in sugarcane juice, location C recorded the highest fungal count (30.33 cfu/mL), followed by locations A and B which had 27.33 & 15.33 cfu/mL respectively as shown in Table 2.

**Table 2:** Total counts associated with sugarcane plant

Type of sample	TC	Location			Mean Fungal count
		A	B	C	
Sugarcane rootlets (cm)	TC	101.33	204.66	214.66	173.55
Sugarcane stem (cm)	TC	44.66	110.66	129.33	94.88
Sugarcane juice (mL)	TC	27.33	15.33	30.33	24.33

*TC* Total count

### Fungal Frequency

#### Rootlets Association

Data in Table 3 show that sugarcane rootlets yielded 781 fungal isolates belonging to 8 fungal species. These are *Alternaria alternate, Aspergillus flavus, Aspergillus niger, Aspergillus parasiticus, Fusarium* spp.*, Penicillium* spp.*, Rhizopus nigricans,* and *Trichoderma harzianum*. On the other hand, the obtained data indicated that *Penicillium* spp. was the most fungal frequency associated with sugarcane rootlets which record 37.13%, followed by *A. niger* (25.61%), *Fusarium* spp. (22.28%), *A. parasiticus* (8.96%), *A. flavus* (2.56%), and each of *A. alternate* and *T. harzianum* which gives 1.54%. *R. nigricans* had less fungal frequency which gave 0.38%.

**Table 3:** Frequency of the isolated fungi associated with sugarcane rootlets

Fungal isolates	Location						Total	
	A		B		C			
	T.C	%	T.C	%	T.C	%	T.C	%
*Alternaria alternata*	NF	–	2	0.26	10	1.28	12	1.54
*Aspergillus flavus*	4	0.51	7	0.90	9	1.15	20	2.56
*Aspergillus niger*	50	6.40	70	8.96	80	10.24	200	25.61
*Aspergillus parasiticus*	16	2.05	28	3.59	26	3.33	70	8.96
*Fusarium* spp.	30	3.84	72	9.22	72	9.22	174	22.28
*Penicillium* spp.	52	6.66	123	15.75	115	14.72	290	37.13
*Rhizopus nigricans*	NF	–	1	0.13	2	0.26	3	0.38
*Trichoderma harzianum*	NF	–	4	0.51	8	1.02	12	1.54
Total	152	19.46	307	39.31	322	41.23	781	100

*T.C* Total count, *N. F* Not found

#### Stems Association

Data in Table 4 indicated that sugarcane stems yielded 427 fungal isolates belonging to 7 species i.e. *A. alternate, A. flavus, A. niger, A. parasiticus, Fusarium* spp.*, Penicillium* spp., and *R. nigricans*. Also, data indicated that *Penicillium* spp. gave a higher fungal frequency associated with sugarcane stems and gave 31.38%, followed by *Fusarium* spp. 29.51%, *A. niger* 13.11%, *A. flavus* 11.24%, *A. parasiticus* 9.84%, and *A. alternate* 3.28% while, *R. nigricans* was less fungal frequency which recorded 1.64%.

**Table 4:** Frequency of the isolated fungi associated with sugarcane stem

Fungal isolates	Location						Total	
	A		B		C			
	T.C	%	T.C	%	T.C	%	T.C	%
*Alternaria alternata*	10	2.34	4	0.94	NF	–	14	3.28
*Aspergillus flavus*	6	1.41	18	4.22	24	5.62	48	11.24
*Aspergillus niger*	4	0.94	22	5.15	30	7.03	56	13.11
*Aspergillus parasiticus*	3	0.70	25	5.85	14	3.28	42	9.84
*Fusarium* spp.	10	2.34	37	8.67	79	18.50	126	29.51
*Penicillium* spp.	34	7.96	57	13.35	43	10.07	134	31.38
*Rhizopus nigricans*	NF	–	3	0.70	4	0.94	7	1.64
Total	67	15.69	166	38.88	194	45.43	427	100

*T.C* Total count, *N.F* Not found

#### Sugarcane Juice Association

Data presented that, sugarcane juice resulted in 219 fungal isolates belonging to 6 fungal species as shown in Table 5. These are *A. alternate, A. flavus, A. niger, A. parasiticus, Fusarium* spp., and *Penicillium* spp. *Penicillium* spp. had the most fungal frequency contaminated sugarcane juice which recorded 37.90% followed by *Fusarium* spp. (29.51%), *A. parasiticus* (22.83%), *A. niger* (12.79%), *Fusarium* spp. (10.50%) and *A. flavus* (8.22%). *A. alternate* has less fungal frequency (7.76%).

**Table 5:** Frequency of the isolated fungi contaminated sugarcane juice

Fungal isolates	Location						Total	
	A		B		C			
	T.C	%	T.C	%	T.C	%	T.C	%
*Alternaria alternata*	10	4.57	4	1.83	3	1.37	17	7.76
*Aspergillus flavus*	5	2.28	3	1.37	10	4.57	18	8.22
*Aspergillus niger*	10	4.57	3	1.37	15	6.85	28	12.79
*Aspergillus parasiticus*	15	6.85	6	2.74	29	13.24	50	22.83
*Fusarium* spp.	2	0.91	14	6.39	7	3.20	23	10.50
*Penicillium* spp.	40	18.26	16	7.31	27	12.33	83	37.90
Total	82	37.44	46	21.00	91	41.55	219	100

*T.C* Total count, *N.F* Not found

### Determination of Aflatoxin Production

**Fig. 1:**
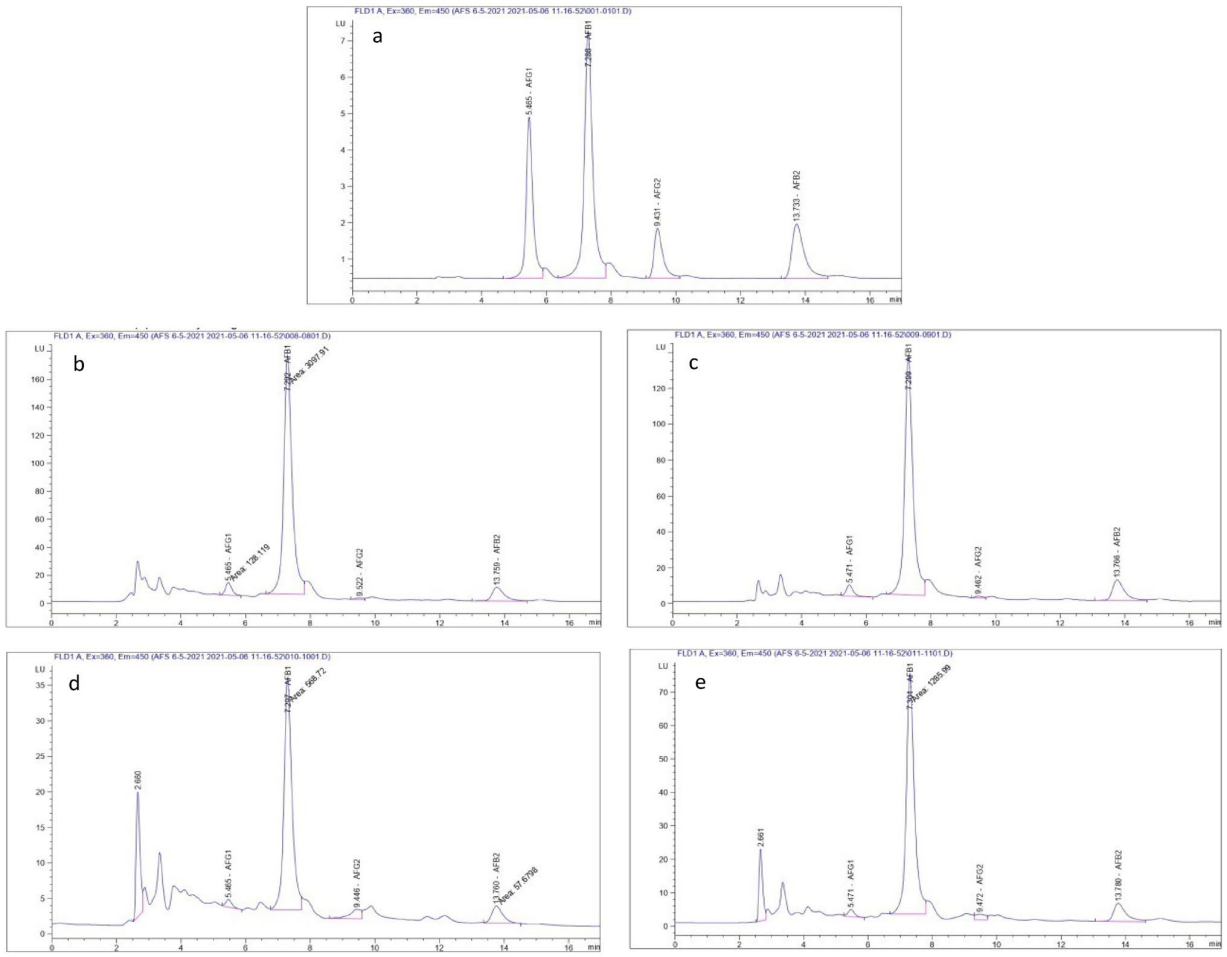
HPLC chromatograms: **a** Standard chromatogram of aflatoxins (B1, B2, G1, and G2), **b** Aflatoxins produced by *A.* *parasiticus* (No 21) isolated from sugarcane stem location B., **c** Aflatoxins produced by *A.* *parasiticus* (No 7) isolated from sugarcane stem location C, **d** Aflatoxins produced by *A.* *parasiticus* (No 5) isolated from sugarcane juice location A., **e** Aflatoxins produced by *A.* *parasiticus* (No 13) isolated from sugarcane juice location C.

Determination of aflatoxins produced by aflatoxigenic fungi isolated from sugarcane resulted that, only four isolates of *A. parasiticus* were found to produce aflatoxins. Two isolates from sugarcane stem from locations B and C samples and two isolates from sugarcane juice from locations A and C samples. On the other hand, a higher aflatoxin quantity was produced by *A. parasiticus* isolates from the sugarcane stem, in which isolate No. 21 from location B samples produced 1434.92 ng/mL)1229.38 AFB1, 98.38 AFG1, 96.61 AFB2 and 10.55 ng/mL AFG2), followed by isolate No. 7 from location C samples which produced 1159.7 ng/mL) 964.74, 73.59, 111.25 and 10.12 ng/mL of AFB1, AFG1, AFB2, and AFG2 respectively(, while *A. parasiticus* isolates from sugarcane juice produced less aflatoxins quantity, whereas isolate No. 13 from location C samples produced 609.55 ng/mL (510.34 AFB1, 23.06 AFG1, 54.87 AFB2, and 21.28 ng /mL AFG2). while isolate No. 5 from location A samples gave 276.95 ng /mL ( 225.69 AFB1, 11.13 AFG1, 21.32 AFB2, and 18.81 ng /mL AFG2), as shown in Table 6 and Fig. 1.

**Table 6:** Determination of aflatoxins produced by aflatoxigenic fungi associated with sugarcane plant

Source	Location	Type of producing fungi	Isolate No	The concentration of Aflatoxin (ng/ml)				Total Aflatoxin (ng/ml)
				AFB_1_	AFG_1_	AFB_2_	AFG_2_	
Sugarcane stem	B	*Aspergillus parasiticus*	21	1229.38	98.38	96.61	10.55	1434.92
	C	*Aspergillus parasiticus*	7	964.74	73.59	111.25	10.12	1159.7
Sugarcane juice	A	*Aspergillus parasiticus*	5	225.69	11.13	21.32	18.81	276.95
	C	*Aspergillus parasiticus*	13	510.34	23.06	54.87	21.28	609.55

### Decontamination of Fungal Association of Sugarcane Juice Using Lemon Juice

Data in Table 7 indicated that lemon juice showed a significant reduction effect on the fungal count of peeled and non-peeled sugarcane juice. On the other hand, the reduction percentage of the fungal count was increased with increasing the concentration of lemon juice used. In which the highest reduction percent of the fungal count was recorded with 20% conc. of lemon on peeled sugarcane juice, which recorded 36.04%, followed by 15% conc. (30.85%), 10% conc. (24.68%), 5% conc. (14.61%), and 9.09% for 2% conc. while in the case of non-peeled sugarcane juice, the highest reduction percent was 21.22% for 20% conc. followed by 15% conc. (18.77%), 10% conc. (17.75%), 5% (15.30%) and 2% (10.20%).

**Table 7:** Decontamination of fungal association of sugarcane juice using lemon juice

Lemon juice Conc	Non-peeled cane juice		Peeled cane juice	
	Mean (cfu/mL)	R%	Mean (cfu/mL)	R%
2%	146.67 ± 7.64^f^	10.20	93.33 ± 1.53^g^	9.09
5%	138.33 ± 4.16^def^	15.30	87.67 ± 1.15^g^	14.61
10%	134.33 ± 1.15^cde^	17.75	77.33 ± 1.15^b^	24.68
15%	132.67 ± 0.58^cd^	18.77	71.00 ± 1.00^ab^	30.85
20%	128.67 ± 3.21^c^	21.22	65.67 ± 0.58^a^	36.04
Control	163.33 ± 15.28^i^		102.67 ± 8.08^h^	
LSD 5%	6.663 B		6.736 A	

Results are mean values of three replicates ± standard deviation. The different letters in each column indicate significant differences at *P* < 05

## Discussion

Sugarcane, (*Saccharum officinarum*), is one of the main crops in the world. It produces over 60% of the world's total sugar requirement, while sugar beet provides the remaining 40% [22]. The sugarcane plant is mostly utilized to produce raw sugar and molasses since it contains a significant amount of sucrose and low fiber content [4]. Sugarcane is frequently contaminated by several moulds which can produce several toxins and cause sugarcane deterioration [9]. A notable decrease in all physical parameters of the naturally infected sugarcane plants (plant length, stem diameter, and juice volume) was observed. Similar results were obtained by Viswanathan and Alexander who found that, severe sugarcane fungal diseases can cause the loss of nearly two-thirds of cane stalks produced in subtropical India [29]. Viswanathan reported that the fungal infection of sugarcane shows a decline of 29 to 83% in cane weight and 24 to 90% in juice extraction [30]. Thangamanil et al. mentioned that the fungal infection reduces the sugarcane juice quality [31]. Gupta et al. reported that the fungal diseases in sugarcane caused a high reduction in the yield parameters like cane girth (5.0–23.4%), cane length (14.4–34.6%) and cane yield (43.1–52.2%) [32]. Xiupeng et al. reported that infected cane stems are relatively small without economic value [33]. Also, Viswanathan found that the fungal infection was responsible for sugarcane deterioration [34].

Isolation of fungi from sugarcane plants from three localities revealed that the highest mean fungal count was recorded in sugarcane rootlets (173.55 cfu/cm), followed by sugarcane stem which recorded 94.88 cfu/cm, while sugarcane juice had the least mean fungal count (24.33 cfu/mL). Similar results were obtained by Kumeda et al. who reported that sugarcane is a suitable host for many saprophytic fungi [35]. Garber isolated *Aspergillus parasiticus* from two previously cropped sugarcane fields, in which the infection of sugarcane stems ranged from 95% in billets prepared for commercial planting to 52% in hand-collected sugarcane stems [36]. Youssef et al. found that the total fungal count of sugarcane bagasse samples in the case of the dilution–plate method (69,720 cfu/g) was higher than those of direct–plate method (828 cfu/20 bagasse segments), and also found that, *Aspergillus* was appeared in a high population (51,700 cfu/g), which represented 74.15% of total count [37]. This microbial contamination of sugarcane may be due to its richness in carbohydrates with a higher water activity which is considered a suitable substrate for fungal growth.

The frequency of the isolated fungi associated with sugarcane rootlets yielded 781 fungal isolates belonging to 8 fungal species (*A. alternate, A. flavus, A. niger, A. parasiticus, Fusarium* spp.*, Penicillium* spp.*, Rhizopus nigricans,* and *Trichoderma harzianum*). *Penicillium* spp. had the most fungal frequency associated with sugarcane rootlets which recorded 37.13%, while *R. nigricans* had less fungal frequency which gave 0.38%. The sugarcane stems yielded 427 fungal isolates belonging to 7 fungal species i. e *Alternaria alternate, Aspergillus flavus, Aspergillus niger, A. parasiticus, Fusarium* spp.*, Penicillium* spp., and *R. nigricans*. On the other hand, *Penicillium* spp. had a higher fungal frequency associated with sugarcane stems and gave 31.38%, while *R. nigricans* had less fungal frequency which recorded 1.64%, while the sugarcane juice yielded 219 fungal isolates belonging to 6 fungal species. These are *A. alternate, A. flavus, A. niger, A. parasiticus, Fusarium* spp. and *Penicillium* spp. *Penicillium* spp. had the most fungal frequency in contaminated sugarcane juice recorded 37.90%, while *Alternaria alternate* had less fungal frequency (7.76%). Similar results were obtained by many investigators. Ahmed et al. isolated 18 different species belonging to 11 different genera of fungi (*Aspergillus flavus*,* A. niger*, *A. terreus, A. fumigatus*, *A. wentii, A. sulphureus, Absidia corymbifera*, *Acremonium* sp., *F. sporotrichoides, Fusarium semitectum, Curvularia lunata, Monilia* sp., *Rhizopus stolonifer*, *R. oryzae, Penicillium* sp., and *Saccharomyces* spp.) from sugarcane juice [5]. Garber reported that the infection of sugarcane stems by *Aspergillus parasiticus* ranged from 95% in billets prepared for commercial planting to 52% in hand-collected sugarcane stems [36]. Romão-Dumaresq et al. and Souza et al. isolated *Aspergillus, Alternaria, Acremonium, Penicillium, Fusarium, Chaetomium, Curvularia*, and *Mucor* from root and rhizosphere of sugarcane plant [38, 39]. Silva et al. identified *Aspergillus parasiticus* as the main species isolated from the sugarcane system [40]. Youssef et al. reported that twenty-five species and four species varieties belonging to 8 genera were isolated from 30 sugarcane bagasse samples. Aspergillus was the most common genus, occurring in (100% of the samples, 88.3% of the total count of fungi), in which, *Aspergillus flavus, A. niger, A. tubengensis,* and *A. phoenicis* were the most dominant species and collected in moderate frequencies of occurrence, while *Acremonium, Fusarium, Curvularia, Penicillium, Mucor* and *Verticillium* were collected and identified in rare frequencies of occurrence [37]. This diversity of fungi may be attributed to several factors such as the temperature, the moisture content, and other characteristics including the pH and type of nutrients and organic content which influence the fungal populations, fungal number, fungal species, and the fungal diversity.

Aflatoxins are secondary metabolites produced mainly by *A. flavus* and *A. parasiticus* under suitable conditions. Determination of aflatoxins produced by aflatoxigenic fungi isolated from sugarcane resulted that, only four isolates of *A. parasiticus* were aflatoxins producers. On the other hand, a higher aflatoxin quantity was produced by *A. parasiticus* isolates from sugarcane stem, whereas isolate No. 21 (Location B) produced 1434.92 ng /mL, and isolate No. 7 (Location C) produced 1159.7 ng /mL, while *A. parasiticus* isolates from sugarcane juice produced less aflatoxins quantity, whereas isolate No. 13 (Location C) produced 609.55 ng /mL, while isolate No. 5 (Location A) gave 276.95 ng /mL. Similar results were obtained by Kumeda et al. and Takahashi et al., They reported that important fungi that produce aflatoxins such as *A. flavus* (18%) and *A. parasiticus* (65%) were detected in sugarcane in Japan [35, 41]. Also, Garber found that aflatoxin-producing fungi that contaminated sugarcane stems ranged from 52 to 95% *A. parasiticus* in hand-collected samples and billets for commercial planting, respectively [36]. Ojo et al. detected aflatoxins B_1_, B_2_, G_1,_ and G_2_, in the examined samples of *S. officinarum* [22]. Hariprasad et al. found that 57 samples of sugarcane juice were taken from Indian local markets, and of those, 22.2% and 19%, respectively, came from Mysore and Mandya. The levels of contamination ranged from 0.5 to 6.5 mg/kg [42]. Abdallah et al. found that only aflatoxin B1 (AFB1) and aflatoxin G1 (AFG1) were found in sugarcane grass and juice meant for human consumption in Upper Egypt, with the highest concentration of 30.6 ng/kg for grass and 2.10 ng/kg for juice, respectively, AFB1 was present in 48% of grass samples and 58% of juice samples. 10% of the grass samples (7.76 ng/kg) and 18% of the juice samples (34 ng /kg) contained AFG1 [7]. Iamanaka et al. reported that *A. parasiticus* was identified as the main aflatoxigenic species isolated from sugarcane and sugarcane soil, and the majority of samples of sugarcane juice (68.5%) were contaminated by aflatoxins, which ranged from 0.4 to 10.2 mg/kg [43]. Silva et al. found that the main aflatoxigenic species found in sugarcane and its byproducts is *A. parasiticus* [40]. The outer fiber layer of the sugarcane stem may be attacked by fungi, especially following insect invasion or other parasites pre- or post-harvest, resulting in the contamination of sugarcane juice with AFs [44, 45].

Because of microbial contamination and enzymatic reactions, sugarcane juice degrades quickly. Synthetic preservatives are typically used to preserve its quality while being stored. Their use has been connected to potential health risks, though. In this sense, various naturally occurring preservatives derived from plants can be used as risk-free substitutes. Lemon is one of the natural preservatives frequently used to preserve fruit juice; it was first added to improve flavor, but it also has antibacterial properties that extend juice's shelf life [46]. Lemon juice showed a significant reduction effect on the fungal count of peeled and non-peeled sugarcane juice, and the reduction percent of the fungal count was increased with increasing the concentration of lemon juice used. In which the highest reduction percent of fungal count was recorded with 20% conc. of lemon on peeled sugarcane juice (36.04%) and 9.09% for 2% conc. while in the case of non-peeled sugarcane juice, the highest reduction percent was 21.22% for 20% conc. and 10.20% for 2% conc. Similar results were obtained by many investigators, Ahmed et al. reported that the addition of lemon juice to sugarcane juice reduced the occurrence of fungi by serial dilution technique [5]. Ramchandran et al. reported that good quality sugarcane juice (100 mL) could be prepared by the addition of lemon (3 mL) as a combination of flavor, color enhancer, and source of citric acid (antioxidant); ginger (0.6 mL) and moringa (10 mL); as flavor enhancer to heat-treated juice, in which lemon was used as a preservative and had a minimum inhibitory concentration (MIC) of 25 mL against 15 identified bacterial strains from fresh sugarcane juice samples, which inhibited the growth of microorganisms during storage [47]. Ramachandran et al. found that lemon juice and *Moringa oleifera* seed extract were added to sugarcane juice at high concentrations during pasteurization to limit microbial development [48]. Rajendran and Bharathidasan reported that the addition of lemon, ginger, and mint to heat-treated sugarcane juice reduces the growth of microorganisms during storage at refrigeration temperatures [49]. Bag et al. reported that about 6.84% of lemon juice is the optimum concentration of preservatives to add to sugarcane juice, while the acidity of sugarcane juice dramatically increased after storage, the lemon was able to drop the pH to 3.0 which had a preservation effect and inhibited the growth of microorganisms [50]. The presence of phenolic components in natural preservatives gives them their antibacterial properties [15].

## Conclusion

The results obtained from the present investigation indicates that, a wide variety of fungal species were present in sugarcane samples from three different localities in Egypt. A number of harmful aflatoxigenic fungi were isolated which produce aflatoxins and caused potential health hazard to human. Moreover, using 20% lemon juice on peeled sugarcane juice exhibited the best antifungal activity, and gave the highest reduction percent of the fungal count in sugarcane juice, so it can serve as an eco-friendly approach for managing fungal contamination in sugarcane juice production and improving its quality, as it is completely natural, easily available, cost efficient, making it valuable natural alternative to chemical preservatives.

## Data Availability

All data included in this study are available upon reasonable request by contacting the corresponding author.
